# Isolation and Characterization of Chicken Dermis-Derived Mesenchymal Stem/Progenitor Cells

**DOI:** 10.1155/2013/626258

**Published:** 2013-08-04

**Authors:** Yuhua Gao, Chunyu Bai, Hui Xiong, Qian Li, Zhiqiang Shan, Linsheng Huang, Yuehui Ma, Weijun Guan

**Affiliations:** ^1^Institute of Animal Sciences, Chinese Academy of Agricultural Sciences, Beijing 100193, China; ^2^College of Wildlife Resources, Northeast Forestry University, Harbin 150040, China

## Abstract

Dermis-derived mesenchymal stem/progenitor cells (DMS/PCs) were isolated from the skin tissue of 16-day-old chick embryos and then characterized by immunofluorescence and RT-PCR. We found that primary DMS/PCs could be expanded for 15 passages. Expression of **β**-integrin, CD44, CD71, and CD73 was observed by immunofluorescence and RT-PCR. Passage 3 DMS/PCs were successfully induced to differentiate into osteoblasts, adipocytes, and neurocytes. The results indicate the potential for multilineage differentiation of DMS/PCs that may represent an ideal candidate for cellular transplantation therapy.

## 1. Introduction

Mesenchymal stem cells (MSCs) were first discovered in bone marrow (BMSCs), which have a strong proliferative capacity and can be differentiated into adipocytes [1, 2], osteoblasts [3–5], myoblasts [6–8], and neurons [9, 10].

However, the proliferation, differentiation, and number of BMSCs are significantly decreased with aging. In addition, because of possible virus infection [11], researchers began to search for MSCs in other tissues. In recent years, MSCs have been found in muscles, amniotic fluid, umbilical cord blood, fat, and other tissues [12–14]. 

The dermis contains mostly differentiated cells including fibroblasts that only participate in scar tissue formation during skin repair [15]. Therefore, the dermis is often regarded as a negative control for studies of stem cells [16]. Following isolation and characterization of BMSCs in the 1990s [2], significant progress has been made in studies of dermis-derived mesenchymal stem/progenitor cells (DMS/PCs), including their separation and culture. Moreover, DMS/PCs can be induced to differentiate into osteoblasts, adipocytes, and ectodermal cell types. Considering the easy accessibility of DMS/PCs, these cells have become an ideal cellular source in tissue engineering. 

Current research of stem cells focuses on humans, mice, rabbits, and other mammals, but little research has been performed on poultry. As an animal model, the chicken possesses abundant dermal tissues. Furthermore, the chicken is an endemic species that is important in the global economy. In this study, we carried out a pilot study on the separation, culture, and differentiation potential of chicken DMS/PCs.

## 2. Materials and Methods

### 2.1. Isolation and Culture of DMS/PCs

Animal experiments were performed in accordance with the guidelines established by the Institutional Animal Care and Use Committee of the Chinese Academy of Agriculture of Sciences.

Dorsal skin tissues were isolated from 30 16-day-old chick embryos. The dermal layer was isolated from the epidermal layer by digestion with 0.25% dispase II (Gibco, Carlsbad, CA, USA) for 1.5–2 h at 37°C. The dermis was cut into approximately 1 cm^2^ pieces and then digested with 0.25% trypsin (Gibco) for about 15 min. Then, the enzymatic activity was neutralized with fetal bovine serum (FBS) (Gibco). The digested tissue was passed through a 200 *μ*m mesh filter and then centrifuged at 1200 r/min for 6 min at room temperature. The supernatant was discarded, and the pellet was re-suspended with an optimized culture medium. The viability of DMS/PCs was determined by trypan blue exclusion. As a result, 1 × 10^4^ cells were yielded from 1 cm^2^ of chick embryo skin. The cell suspension was seeded in six-well plates and incubated at 37°C with 5% CO_2_. After 48 h of culture, the cells were washed twice with PBS to remove nonadherent cells. At 70–80% confluence, the cells were passaged with 0.25% trypsin. Generally, after 3-4 passages, the cells were homogenous.

### 2.2. Optimization of Cell Culture Systems for DMS/PCs

DMS/PC culture at passage 3 was assessed in three culture systems: culture system I (L-Dulbecco's modified Eagle's medium (DMEM) supplemented with 10% FBS), culture system II (L-DMEM supplemented with 2 mM L-glutamine, 1 mM sodium pyruvate, and 10% FBS), and culture system III (L-DMEM supplemented with 20 ng/mL epidermal growth factor (EGF), 20 ng/mL basic fibroblast growth factor (bFGF), 10% FBS, 2 mM L-glutamine, and 1 mM sodium pyruvate). Cells were harvested and reseeded in six-well plates at 5 × 10^4^ cells/well. The cells were cultured further and the generation time in each culture system was counted three times. Culture system III was subsequently used to culture DMS/PCs.

### 2.3. Markers of DMS/PCs

DMS/PCs were fixed in 4% paraformaldehyde for 15 min and then washed three times in PBS (5 min each). Cells were permeabilized with 0.2% Triton X-100 for 15–20 min and then washed three times (5 min each) in PBS. The cells were blocked with 10% normal goat serum (Santa Cruz Biotechnology, Santa Cruz, CA, USA) for 30 min and then incubated at room temperature for 1 h in 3% bovine serum albumin (BSA) containing the following antibodies: mouse anti-*β*-integrin (1 : 100; Abcam, Cambridge, MA, USA), mouse anti-CD44 (1 : 200; Abcam), mouse anti-nestin (1 : 200; Abcam), rabbit anti-synaptophysin (SYP) (1 : 100; Bioss, Beijing, China), rabbit anti-CD71 (1 : 200; Bioss), rabbit anti-CD73 (1 : 200; Bioss), rabbit anti-neurofilament (NF) (1 : 200; Bioss), or goat anti-*β*-III tubulin (1 : 200; Santa Cruz Biotechnology). Then, the cells were washed three times (10 min each) with PBS and then incubated in PBS containing secondary antibodies at 37°C for 1 h. Secondary antibodies were Cy5.5-conjugated goat anti-mouse and donkey anti-rabbit IgGs, and fluoroisothiocyanate-conjugated goat anti-rabbit and donkey anti-goat IgGs (Santa Cruz Biotechnology).

Cells were examined under a TE-2000-E inverted fluorescence microscope (Nikon, Yokohama, Kanagawa Japan). Cells were counterstained with DAPI (Sigma-Aldrich, St. Louis, MO, USA).

### 2.4. RT-PCR

RNA was isolated from cells using Trizol reagent (Invitrogen). cDNA was synthesized using a reverse transcription system (Takara, Dalian, Liaoning, China) and amplified by PCR using specific primers (Table 1). PCR products were visualized by 2% agarose gel electrophoresis.

### 2.5. Adipogenic Differentiation of DMS/PCs

Cells were divided into two groups: induced and control groups. At 50–60% confluence, cells in the induced group were incubated in adipogenic medium containing 1 mM dexamethasone (Sigma), 0.5 mM isobutyl-methylxanthine (IBMX; Sigma), and 10 *μ*g/mL insulin (Sigma). Cells in the control group were cultured in complete medium without any inducers. After 2 weeks of differentiation, the cells were stained with oil red O to assess intracellular lipid accumulation. RNA was also isolated for RT-PCR analysis.

### 2.6. Osteogenic Differentiation of DMS/PCs

Passage 3 cells were seeded in six-well plates at 1 × 10^4^ cells/well. The cells were also divided into inducted and control groups. At 50–60% confluence, cells in the induced group were cultured in osteogenic medium containing 0.5 mM dexamethasone (Sigma), 10 mM *β*-glycerophosphate (Sigma), and 50 *μ*g/mL vitamin C. Cells in the control group were cultured in complete medium without any inducers. Media were changed every 2 days. After 2 weeks of differentiation, the capacity for calcium node formation was detected by alizarin red staining and osteoblast-specific gene expression was analyzed by RT-PCR. 

### 2.7. Neurogenic Differentiation of DMS/PCs

Cells were seeded and divided into the two groups as described above. Neural-like cell differentiation was accomplished in L-DMEM supplemented with 10% FBS, 1 *μ*M all-trans-retinoic acid, and 100 *μ*M 2-mercaptoethanol (Sigma) [17]. After 10 days, the cells were harvested and neural-specific marker expression was detected by immunofluorescence and RT-PCR.

## 3. Results

### 3.1. Isolation, Culture, and Morphology of DMS/PCs

**Figure 1:**
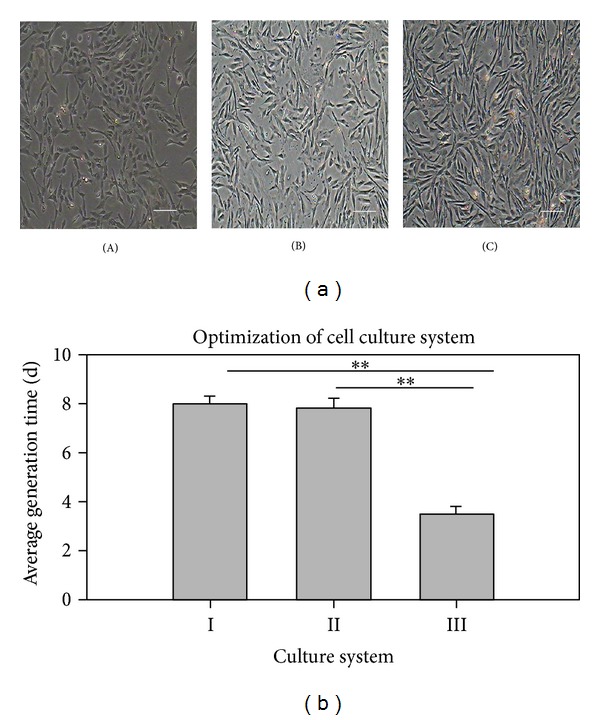
(a) Morphology of primary and subcultured DMS/PCs. (A) After 24 h of primary culture, DMS/PCs exhibited a shuttle shape with clear boundaries and grew slowly. (B) After 5 days of primary culture, DMS/PCs showed polygonal and long shuttle shapes, most of which had protrusions, and gradually reached confluency. (C) Passage 3 DMS/PCs were fibroblast-like and homogeneous (scale bar: 100 *μ*m). (b) Comparison of cell proliferation in different culture systems. Culture systems III was suitable for DMS/PC proliferation. Data are expressed as the means ± S.D. of triplicates (**P < 0.01).

Primary cells isolated from the dermis adhered to the culture plates and began to elongate after 24 h (Figure 1(a)-A). After about 5 days, the cells exhibited a fibroblast-like morphology (Figure 1(a)-B) and grew to 80–90% confluence (Figure 1(a)-C). 

### 3.2. Optimization of DMS/PC Culture

There was no significant difference between culture systems I and II (*P* > 0.05). The generation time was about 8 days for both systems. Culture system III and the other culture systems were significantly different and resulted in a generation time of about 3 days (*P* < 0.01) (Figure 1(b)). These results indicated that EGF and bFGF promote DMS/PC proliferation, and culture system III is suitable for expansion of DMS/PCs.

No obvious morphological differences were observed among passages, and the characteristics of the cells were stable after passaging. The cells were cultured to passage 16 and showed the representative appearance of senescence, such as blebbing and karyopyknosis in most cells. Moreover, cells cultured for more than 16 passages became detached from the plates. 

### 3.3. Characterization of DMS/PCs 

#### 3.3.1. Markers of DMS/PCs

**Figure 2:**
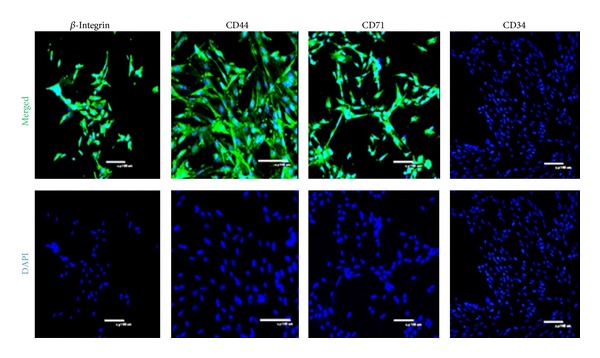
Surface antigen characterization of DMS/PCs at different passages. DMS/PCs expressed numerous surface markers such as *β*-integrin, CD44, CD71, and CD73 but not CD34 (a hematopoietic marker). Immunofluorescence showed that *β*-integrin and CD44 were positive, while CD34 was negative (scale bar: 100 *μ*m).

**Figure 3:**
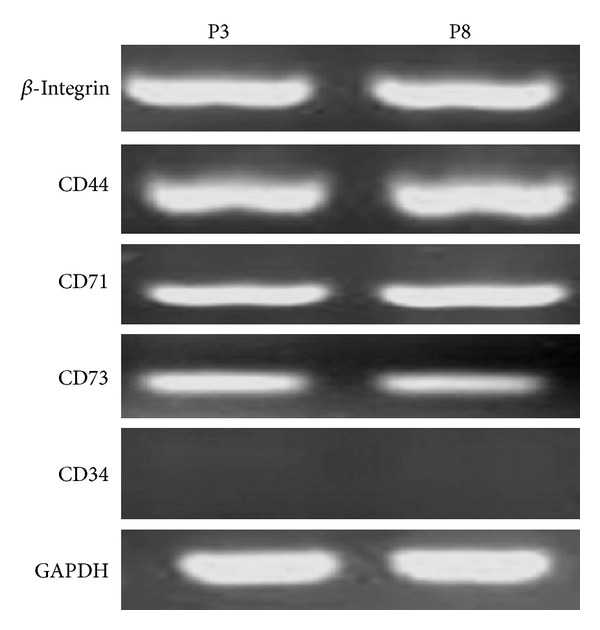
RT-PCR assays RT-PCR analysis showed that different passages DMS/PCs expressed *β*-integrin, CD44, CD71, and CD73. CD34 expression was negative. GAPDH served as the internal control.

We detected markers of DMS/PCs by immunofluorescence and RT-PCR. The immunofluorescence (Figure 2) and RT-PCR (Figure 3) results showed that DMS/PCs expressed *β*-integrin, CD44, CD71, and CD73 but did not express CD34 (a hematopoietic cell marker). There were no apparent differences in these markers at different passages.

### 3.4. Adipogenic Differentiation of DMS/PCs

**Figure 4:**
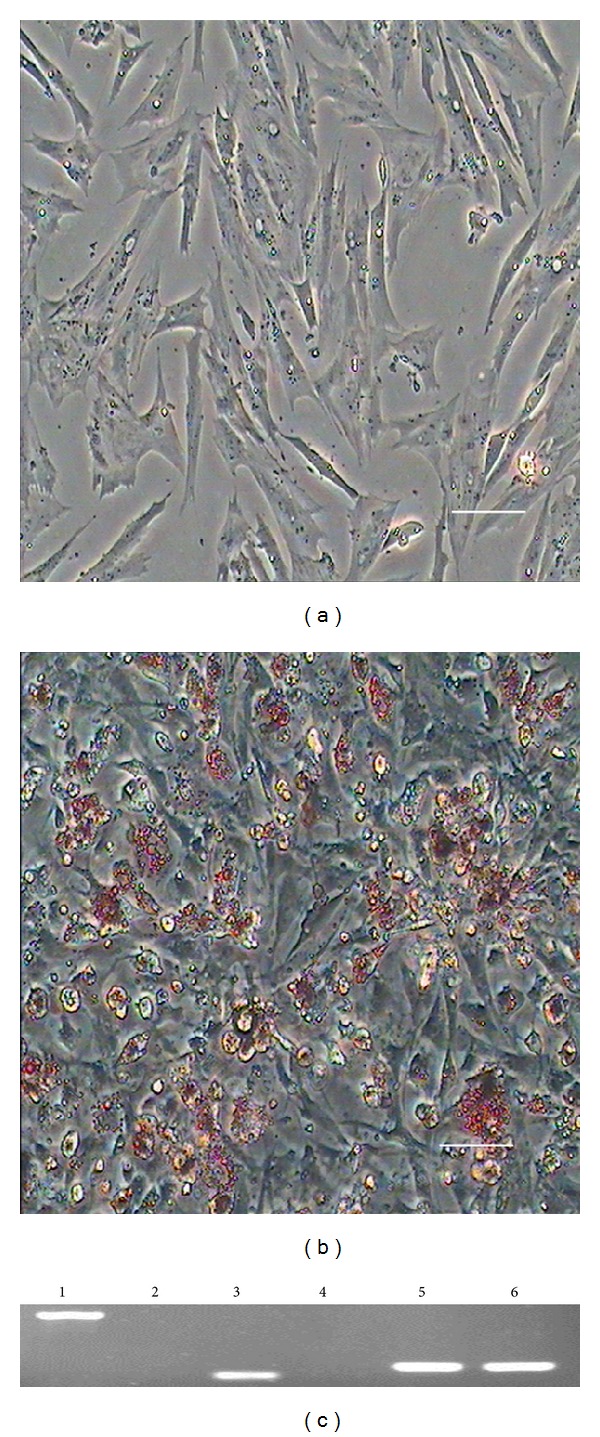
Adipogenic differentiation of DMS/PCs. (a) As a negative control, cells cultured in complete medium showed no changes in morphology and were negative for oil red O staining. (b) After induction for 7 days, DMS/PCs became fibroblast-like to oblate and formed many intracellular lipid droplets. Lipid droplets were stained with oil red O (scale bar: 100 *μ*m). (c) Expression of adipocyte-specific genes LPL and PPAR-*γ* was detected by RT-PCR in the induced group after induction for 14 days. Adipocyte-specific genes were not expressed in the control group. Lane 1: LPL was positive in the inducted group; lane 2: LPL was negative in the control group; lane 3: PPAR-*γ* was positive in the inducted group; lane 4: PPAR-*γ* was negative in the control group; lanes 5-6 GAPDH served as the internal control.

Adipogenic differentiation of DMS/PCs was demonstrated by oil red O staining [18]. After incubation in adipogenic medium for 7 days, DMS/PCs changed from a shuttle shape to an oblate shape and contained many intracellular lipid droplets. As differentiation progressed, the number of lipid droplets increased and aggregated to form larger droplets (Figure 4(b)). As a negative control, cells cultured in complete medium were negative for oil red O staining (Figure 4(a)). 

After induction, RT-PCR results showed that the cells expressed adipocyte-specific genes peroxisome proliferator-activated receptor-*γ* (PPAR-*γ*) and lipoprotein lipase (LPL), whereas these genes were not expressed in the control group (Figure 4(c)).

### 3.5. Osteogenic Differentiation of DMS/PCs 

**Figure 5:**
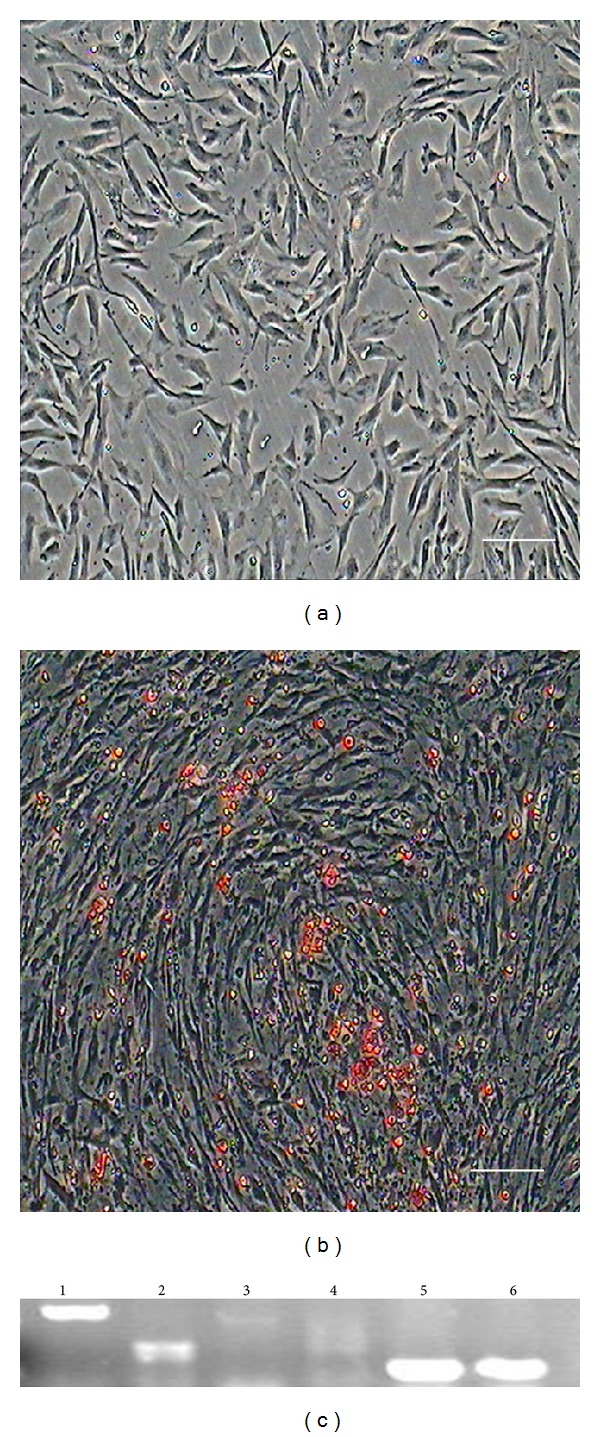
Osteogenic differentiation of DMS/PCs. (a) Control cells. (b) After induction in osteogenic medium for 14 days, the cells changed from fusiform to triangular in shape and were positive for alizarin red staining. Calcified nodules increased in number and became larger during induction. After about 14 days, the nodules were observed by alizarin red staining. Cells cultured in complete medium showed no morphological changes and were negative for alizarin red staining (scale bar: 100 *μ*m). (c) After induction for 14 days, RT-PCR revealed the expression of osteoblast-specific genes ALP and OPN in the induced group, whereas these genes were not expressed in the control group. Lane 1: AKP was positive in the inducted group; lane 2: OPN was positive in the inducted group; lane 3: AKP was negative in the control group; lane 4: OPN was negative in the control group; lanes 5 and 6: GAPDH served as the internal control.

After incubation in osteogenic medium for 7 days, DMS/PCs showed obvious morphological changes. After 14 days of differentiation, the cells became aggregated and formed mineralized nodules that were stained with alizarin red. In addition, the number and size of nodules were increased (Figure 5(b)), whereas control cells showed no such effects (Figure 5(a)). 

Osteogenic differentiation of DMS/PCs was also analyzed by RT-PCR. Osteogenic-specific genes alkaline phosphatase (AKP) and osteopontin (OPN) were expressed in the induced group but not in the control group (Figure 5(c)).

### 3.6. Neurogenic Differentiation of DMS/PCs

**Figure 6:**
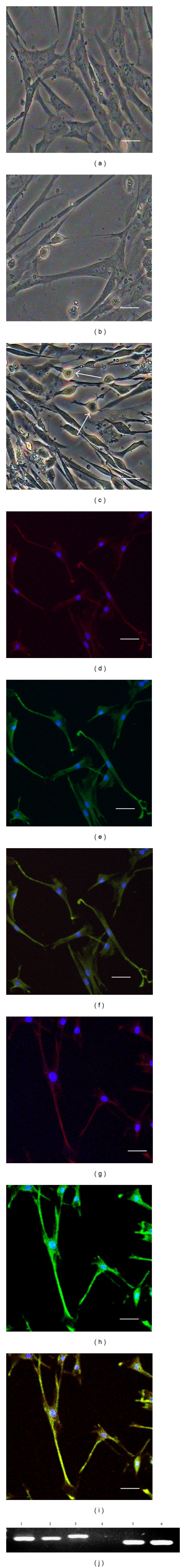
Neural differentiation of DMS/PCs. (a) Control cells. (b) After 1 week of induction, neural-like cells with a multipolar spindle-like shape were observed. (c) After 2 weeks of induction, neural-like cells were observed as indicated by the arrow. (d–i) Double immunofluorescence staining showed that (d) nestin (red) and (e) *β*-III tubulin (green) were positive in the induced cells. (f) Merged image of d and e. (g) NF (red) and SYP (h) were positive in the inducted group. (i) Merged image of g and h. Cells were counterstained with DAPI (blue) (scale bar: 100 *μ*m). (j) After induction for 14 days, expression of neural-specific genes nestin and NF was detected by RT-PCR in the induced group, whereas these genes were not expressed in the control group. Lane 1: nestin was positive in the inducted group; lane 2: nestin was positive in the control group; line 3: NF was positive in the inducted group; lane 4 NF was negative in the control group; lanes 5 and 6: GAPDH served as the internal control.

After incubation in neural differentiation medium for 14 days, DMS/PCs exhibited elongated cell bodies with neurites (Figures 6(b) and 6(c)). There were no obvious morphological changes in the control group (Figure 6(a)). Moreover, immunofluorescence demonstrated that cells in the inducted group expressed neural cell markers nestin (Figure 6(d)), *β*-III tubulin (Figure 6(e)), NF (Figure 6(g)), and SYP (Figure 6(h)). RT-PCR analysis demonstrated expression of the nestin gene in both inducted and control groups, but the relative expression level in the inducted group was significantly higher than that in the control group (Figure 6(j), lanes 1 and 2). In addition, the NF gene was expressed in the induction group (Figure 6(j), lanes 3 and 4). These results indicate that DMS/PCs can differentiate into neurocytes [19].

## 4. Discussion

In this study, DMS/PCs were successfully isolated from the dermis of 16-day-old chick embryos. Obvious differences in cell viability were observed between cells isolated from 16-day-old and 21-day-old embryos (data not show), indicating that younger animals are more suitable and the conditions to separate the dermis should be considered carefully.

The markers of DMS/PCs resemble those of BMSCs. Both cell types express some surface markers of MSCs. We examined the expression of *β*-integrin, CD44, CD71, and CD73 by immunofluorescence and RT-PCR. *β*-integrin is an integrin unit associated with very late antigen receptors. It is involved in cell adhesion and recognition in various biological processes including embryogenesis, hemostasis, tissue repair, immune responses, and metastatic diffusion of tumor cells. CD44 is a cell surface glycoprotein involved in cell-cell interactions, adhesion, and migration. This protein participates in a variety of cellular functions including lymphocyte activation, recirculation and homing, hematopoiesis, and tumor metastasis. CD71 is a member of the transferrin receptor family that is required for the import of iron into cells and is regulated in response to intracellular iron concentrations. Low iron concentrations increase the levels of transferrin receptors to increase iron intake into cells. Thus, the transferrin receptor maintains cellular iron homeostasis. CD73, also known as ecto-5′-nucleotidase, is an enzyme used as a marker of lymphocyte differentiation [20].

Multilineage differentiation of stem cells is the most notable characteristic for homotransplantation. Because of easy accessibility, DMS/PCs have become an ideal cell source in tissue engineering. In vivo, the development and function of tissue stem cells are related to transcription factors and extracellular signals [21]. However, in vitro, the mechanisms of differentiation are unclear. In our study, we differentiated chicken DMS/PCs into osteoblasts, adipocytes, and neurocytes, and then examined relevant gene expression of these cell types. The results showed that different induction factors affect the differentiation of DMS/PCs. In addition, DMS/PCs originating from mesoblastema can be induced to differentiate into mesodermal and ectodermal cells. The homotransplantation feature of DMS/PCs, together with their putative multipotency and ease of procurement, suggests that these cells are an excellent choice for many tissue engineering strategies and cell-based therapies. The chick embryo is a classic model of vertebrate developmental biology, which has been used for many decades [22]. Although the multi-lineage differentiation of DMS/PCs was successful in vitro, there are many drawbacks for the use of these cells in tissue regeneration in vivo, such as a higher decline rate and unstable phenotype. Therefore, more consideration may be needed for further research.

## 5. Conclusions

In this study, we isolated DMS/PCs from the dermis of 16-day-old chick embryos and then examined their ability to expand and differentiate in vitro. These results have implications for the potential utility of the dermis as a source of stem cells for regenerative medical therapies.

## Figures and Tables

**Table 1:** Primer sequences used in RT-PCR assay.

Gene	Primer sequence	Tm (°C)	Fragment (bp)
*β*-integrin	F 5′ GAACGGACAGATATGCAACGG 3′	60	300
	R 5′ TAGAACCAGCAGTCACCAACG 3′		
CD44	F 5′ CATCGTTGCTGCCCTCCT 3′	58	290
	R 5′ ACCGCTACACTCCACTCTTCAT 3′		
CD71	F 5′ CCCAGGCTTCCCTTCGT 3′	56	305
	R 5′ GGGCTCCAATCACAACATAC 3′		
CD73	F 5′ TCCCGTTTCAAGGGTCAG 3′	52.6	310
	R 5′ GTCCTCCAATAACAACATCCACTC 3′		
AKP	F 5′ TTACCTCTGCGGCGTCAA 3′	59.1	556
	R 5′ CCTGTCCAGCTCATACACCATA 3′		
OPN	F 5′ CAGAACAGCCGGACTTTC 3′	51	227
	R 5′ CTTGCTCGCCTTCACCAC 3′		
PPAR-*γ*	F 5′ CTGTCTGCGATGGATGAT 3′	47.3	199
	R 5′ AATAGGGAGGAGAAGGAG 3′		
LPL	F 5′ AGTGAAGTCAGGCGAAAC 3′	48.7	477
	R 5′ ACAAGGCACCACGATT 3′		
NF	F 5′ CCAGTCCGACCACAACAT 3′	56	231
	R 5′ TCCTGGTACTCCCTCAAAT 3′		
Nestin	F 5′ CAACGAGCCTACATTGCTAA 3′	56	289
	R 5′ CTCATCTGGGAACTCACATTC 3′		
GAPDH	F 5′ TAAAGGCGAGATGGTGAAAG 3′	53	244
	R 5′ ACGCTCCTGGAAGATAGTGAT 3′		
